# Sustained Intermittent Hypoxemia Induces Adiponectin Oligomers Redistribution and a Tissue-Specific Modulation of Adiponectin Receptor in Mice

**DOI:** 10.3389/fphys.2019.00068

**Published:** 2019-02-08

**Authors:** Mélany Pierard, Alexandra Tassin, Stéphanie Conotte, Karim Zouaoui Boudjeltia, Alexandre Legrand

**Affiliations:** ^1^Laboratory of Respiratory Physiology, Pathophysiology and Rehabilitation, Research Institute for Health Sciences and Technology, University of Mons, Mons, Belgium; ^2^Laboratory of Experimental Medicine (ULB 222), Medicine Faculty, CHU de Charleroi, Université Libre de Bruxelles, Brussels, Belgium

**Keywords:** animal model, hypoxemia, adiponectin, oligomer, hypoxia, COPD

## Abstract

**Introduction:** Hypoxemia is a critical component of several respiratory diseases and is known to be involved in the processes underlying co-morbidities associated to such disorders, notably at the cardiovascular level. Circulating level of Adiponectin (Ad), known as a metabolic regulator and cardio-protective hormone was previously suggested to be reduced by hypoxia but consequences of such variation are unclear. The evaluation of the specific effect of hypoxemia on Ad forms and receptors could improve the understanding of the involvement of Ad axis in hypoxemia-related diseases.

**Methods:** Ad-pathway components were investigated in a murine model of sustained intermittent hypoxemia (FiO_2_ 10%, 8 h/day, 35 days).

**Results:** Sustained intermittent hypoxemia (SIH) induced a redistribution of Ad multimers in favor of HMW forms, without change in total plasmatic level. Mice submitted to hypoxia also exhibited tissue-specific modification of adiporeceptor (AdipoR) protein level without mRNA expression change. A decreased AdipoR2 abundance was observed in skeletal muscle and heart whereas AdipoR1 level was only reduced in muscle. No change was observed in liver regarding AdipoR. Lipid profile was unchanged but glucose tolerance increased in hypoxemic mice.

**Conclusion:** Sustained intermittent hypoxemia, *per se*, modify Ad oligomerization state as well as AdipoR protein abundance in a tissue-specific way. That suggests alteration in Ad-dependant pathways in pathological conditions associated to SIH. Investigation of Ad-pathway components could therefore constitute useful complementary criteria for the clustering of patients with hypoxemia-related diseases and management of co-morbidities, as well as to develop new therapeutic strategies.

## Introduction

Adiponectin (Ad), a 30-kDa protein mainly secreted by adipose tissue, is known for its anti-inflammatory, anti-atherogenic, and anti-diabetic effects on multiple target tissues such as liver, heart, and muscle (Yano et al., 2008; Yamauchi and Kadowaki, 2008; Tian et al., 2012). Post-translational modifications of Ad are required for its biological activity and its secretion in the blood stream. Ad exists in three different forms in the circulation: low (LMW), medium (MMW), and high molecular weight (HMW) forms (Wang et al., 2008; Yamauchi and Kadowaki, 2008). HMW forms are considered as the most biologically active forms through their more potent ability to activate AMPK and increase insulin sensitivity (Wang et al., 2008; Yamauchi and Kadowaki, 2013). Ad exerts its beneficial effects on glucose and lipid metabolism via two receptors: AdipoR1 and AdipoR2 (Yamauchi et al., 2007, 2014). These receptors share 67% amino acid identity and possesses seven transmembrane domains. These receptors are ubiquitously expressed, with the most important expression in skeletal muscle and liver for AdipoR1 and AdipoR2, respectively (Yamauchi and Kadowaki, 2013; Yamauchi et al., 2014; Wang et al., 2017). Adiporeceptor (AdipoR) were shown to activate different signaling pathways. Indeed, AdipoR1 is associated to AMPK activation, whereas AdipoR2 is involved in PPAR-α activation (Yamauchi et al., 2007).

In previous *in vitro* studies, Ad expression was shown to be modulated upon hypoxic conditions. Indeed, as hypoxia was suggested to appear in adipose tissue in case of obesity, some scientists have evaluated Ad expression in adipocytes exposed to hypoxia *in vitro*. They observed that hypoxia reduced Ad expression at mRNA and protein levels (Hosogai et al., 2007; Ye et al., 2007; Guo et al., 2017; Priyanka et al., 2017). In another study, a reduced secretion of total Ad and HMW forms was observed in 3T3-L1 adipocytes exposed to intermittent hypoxia (Magalang et al., 2009). In rodents, exposure to a chronic intermittent hypoxia reduced serum Ad levels (Fu et al., 2015; Pan et al., 2015) as well as Ad mRNA expression in white adipose tissue (Fu et al., 2015). A reduced Ad plasmatic (Ad_pl_) level was also observed in rats exposed to continuous neonatal hypoxia for 8 weeks (Chaiban et al., 2008). However, He et al. (2014) reported that the intensity of the effect on Ad_pl_ level depends on the pattern of hypoxemia. Indeed, intermittent hypoxemia (IH: FiO_2_: 5%, 30 s hypoxemia/90 s normoxia, 8 h/day for 8 weeks) and sustained intermittent hypoxemia (SIH: FiO_2_: 10%, 8 h/day for 8 weeks) both reduced Ad_pl_ level compared with normoxic rats but a higher decrease was observed in the IH model (He et al., 2014). The authors also mentioned that these different patterns of hypoxemia could induce similar effects through distinct signaling pathways. This emphasizes that divergent patterns of hypoxia exposure have to be applied to study the effect of this component in different pathological contexts. Indeed, intermittent hypoxemia is a major factor contributing to pathophysiological processes of obstructive sleep apnea (Dewan et al., 2015), whereas continuous hypoxemia is a hallmark feature of people living at high altitude or of patients with severe Chronic Obstructive Pulmonary Disease (COPD) (Lewis and O’Halloran, 2016). Indeed, progression of COPD is associated to a lung function decline leading to an increased risk of hypoxemia in these patients (Kent et al., 2011; Tantucci and Modina, 2012). Before the development of a persistent hypoxemia, the dysregulation of the central respiratory drive contributes to the occurrence of a nocturnal desaturation in COPD patients (Kent et al., 2011; Lewis and O’Halloran, 2016). An exercise-induced desaturation is also observed in a large cohort of COPD patients (van Gestel et al., 2012). Although 5 and 61% of COPD patients exhibited nocturnal or exercise-induced desaturation, respectively (Lewis et al., 2009; van Gestel et al., 2012), the relationship between this pattern of hypoxemia and Ad production and effects remains unclear. The use of an *in vivo* model of SIH seems appropriate to evaluate the specific effect of moderate hypoxemia on Ad pathways in this pathological context. Indeed none of the previous studies evaluated the specific effect of hypoxemia on Ad signaling pathway, whereas a modulation of AdipoR abundance as well as Ad multimer (Ad_mer_) distribution could considerably modify the beneficial effect of Ad on oxidative stress, inflammation, and metabolism. Indeed, Yamauchi et al. (2007) observed that disruption of AdipoR1 and AdipoR2 expression counteracts Ad’s effects and enhanced tissue triglyceride content, insulin resistance, and glucose intolerance.

In the present study, by using a reductionist murine model, we evaluated the specific effect of a SIH on Ad_pl_ level, Ad_mer_ distribution, and AdipoR abundance in several tissues as well as associated systemic metabolic abnormalities.

## Materials and Methods

### Ethics Statement

All procedures met the Belgian national standard requirements regarding animal care and were conducted in accordance with the Ethics and Welfare Committee of the University of Mons. The protocol was approved by the Ethics and Welfare Committee of the University of Mons (reference number LE020/02).

### Animals

Mice were housed in cages with *ad libitum* access to water and food and were maintained at 35–40% relative humidity and a temperature of 20–23°C in a 12:12 h light–dark cycle. At 6 weeks of age, male C57BL6J mice bred in our animal facility (accreditation number LA1500022) were randomly assigned into two experimental groups: mice submitted to sustained intermittent hypoxemia (SIH, *n* = 13) and control mice (Ctl, *n* = 13). Mice were exposed to sustained intermittent hypoxia (10% FiO_2_, 8 h/day, 7 d/week during light cycle) for 35 days in a device previously developed and validated (Chodzyński et al., 2013). Normoxic mice were exposed to ambient air in a similar cage and placed nearby SIH mice to reproduce similar noises. Food intake, and body weight were measured once a week during the 5-week exposure period. Mice were housed individually and food intake was evaluated by measuring food weight every week. The day following the end of the protocol, mice were sacrificed, blood and tissues were collected for RT-qPCR, ELISA and Western blot analysis. Hematocrit measurement was performed on blood sample by using a hemocytometer.

### Glucose Tolerance Test

A glucose tolerance test (GTT) was performed at day 0, 1, 7, 14, 21, 28, 35 as described in Pierard et al. (2016). Briefly, an intraperitoneally administration of a dose of 2 g/kg body weight of D-glucose (Roth, Karlsruhe, Germany) to fasting animals was realized. Blood glucose level was measured 0, 30, 60, and 120 min after glucose injection using a One Touch^®^Vita^®^glucometer (Zug, Switzerland).

### Triglyceride and Cholesterol Measurement

Triglyceride and cholesterol plasmatic levels were assessed by an enzymatic colorimetric method according to the manufacturer’s instructions (triglycerides: 1 5710 99 10 021, cholesterol: 1 1300 99 10 021, Sopachem Diagnostics, Belgium).

### RNA Extraction – Reverse Transcription and Real-Time PCR

The total RNA from frozen muscle, liver and heart was extracted using the miRNeasy Micro Kit (Qiagen^®^, Hilden, Germany) according to the manufacturer’s instructions. The same amount of RNA was reverse transcribed into cDNA with PrimeScript^TM^ RT reagent Kit with gDNA Eraser (Takara, Japan). This reverse transcription kit included a DNase I digestion step to avoid genomic DNA contamination. The qPCR was performed with Lightcycler 480 Real-Time PCR II (F. Hoffmann Roche^®^, Ltd., Basel, Switzerland). The cycling conditions were as follows: 30 s at 95°C, 40 cycles of 20 s at 60°C, and 15 s at 65°C. All samples were run in duplicate. The primers used for AdipoR and RPLP0 are detailed in supporting informations (Supplementary Table S1). The target gene cycle threshold (Ct) was normalized to the expression of the housekeeping gene RPLP0, and gene expression was calculated using the δCt method.

### ELISA

The Ad and leptin plasmatic concentration were measured by using the mouse Ad/Acrp30 Quantikine ELISA Kit and the mouse Leptin Quantikine ELISA Kit, according to the manufacturer’s instructions (Ad: MRP300; Leptin: MOB00, R&D Systems, Minneapolis, MN, United States).

### Western Blot

The relative amounts of LMW, MMW, and HMW Ad_mer_ as well as AdipoR protein level were evaluated as previously described in Pierard et al. (2016). Briefly, Ad_mer_ proportion was determined using a non-denaturing PAGE–SDS followed by a Western blot. 5 μl of plasma diluted to contain 5 μg/ml of Ad was loaded onto a 6% polyacrylamide gel in the presence of SDS. The AdipoR protein level was evaluated on frozen heart, liver, and skeletal muscle tissue (gastrocnemius). 50 μg of total protein extracts were loaded on 12% gel and transferred on a nitrocellulose membrane (Millipore, Darmstadt, Germany). Red Ponceau dye was used for protein detection and to confirm the quality of protein transfer. After blocking with 5% fat-free dry milk-TBS, the membranes were incubated with a rabbit polyclonal primary antibody directed against Ad (Ab85827, 1:1000, Abcam, Cambridge, United Kingdom), against AdipoR1 (1:1000) or AdipoR2 (1:750) (AdipoR12-A, AdipoR22-A; Alpha Diagnostic, San Antonio, TX, United States). Primary antibodies were detected with an appropriate horseradish peroxidase-labeled secondary antibody (1:5000, Sigma-Aldrich, St. Louis, MO, United States) and revealed with the ECL^TM^ Western Blotting Detection kit (GE Healthcare, Little Chalfont, United Kingdom) or Femto (Thermo Fisher Scientific, Waltham, MA, United States). The immunoreactive bands were then submitted to a densitometric analysis using the Image J software. The drastic alteration of metabolic and vascular pathways induced by hypoxia necessitated the use of a loading control other than traditionally used actin, β-tubulin, or GAPDH controls (Yamaji et al., 2003; Bakhashab et al., 2014; Zieseniss, 2014; Parker et al., 2017). While RPLP0 is not modulated by hypoxia and was used in RT-qPCR experiment as a housekeeping gene, the molecular weight of its protein is too close of that of AdipoR. Moreover, the protein abundance of RPLP0 vary depending on the tissue, making it difficult to detect in all tissues. Given that no perfect loading control exists in hypoxic condition, equal loading was confirmed using Ponceau staining. Such normalization was previously realized for AdipoR detection (Mullen et al., 2009; Matyal et al., 2014).

### Statistical Analysis

The statistical analyses of body weight, food intake and glucose tolerance were performed using a Mann–Whitney rank sum test. Hematocrit, HMW proportion, Ad and leptin plasmatic levels as well as triglyceride, and cholesterol levels were assessed using a Student’s *t*-test. A Mann–Whitney rank sum test was used to determine the statistical significance for mRNA expression and protein level of AdipoR1/2 in muscle, heart and liver. Differences were considered statistically significant at a *P*-value < 0.05. All data are represented as mean ± SEM or boxplot (5th and 95th percentile) for parametric or non-parametric statistical tests, respectively.

## Results

### Sustained Intermittent Hypoxemia Induced a Secondary Erythrocytosis in Mice

**FIGURE 1:**
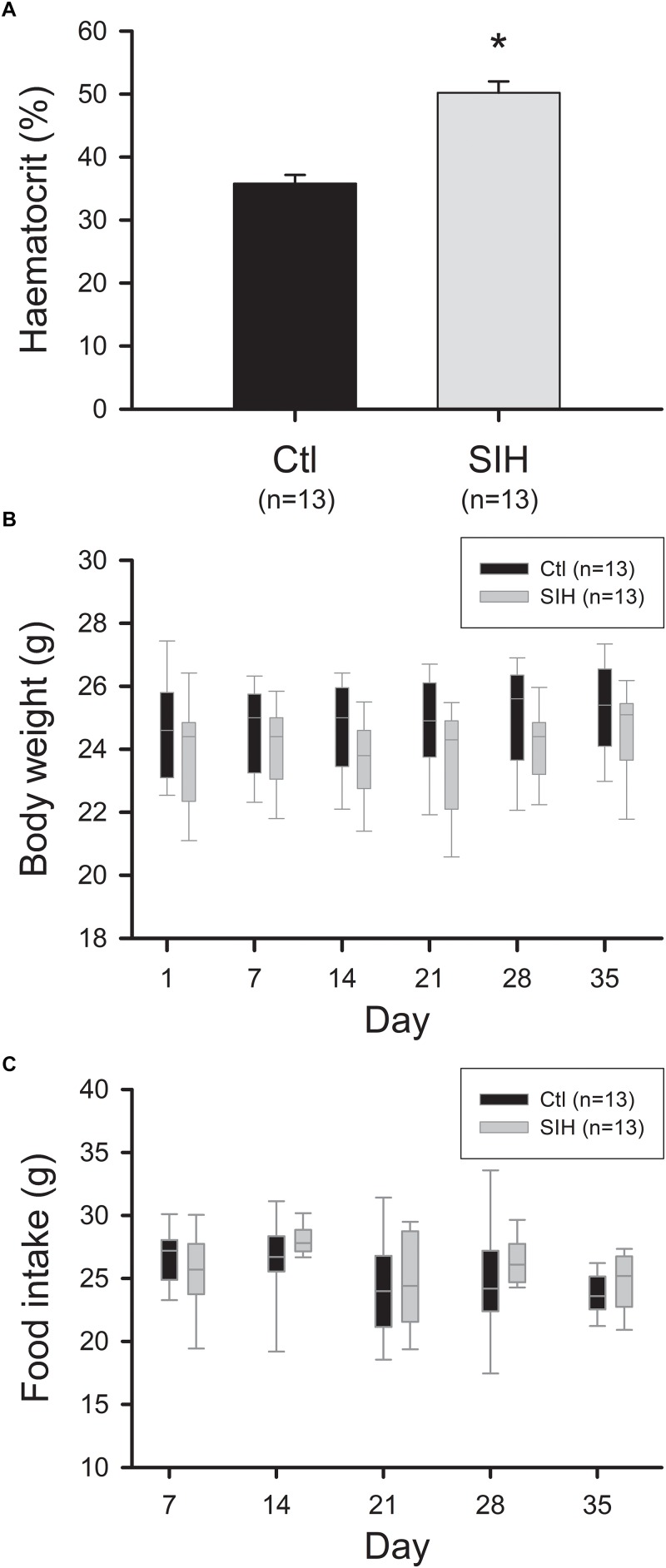
Effect of hypoxemia on hematocrit, body weight and food intake. **(A)** Hematocrit level. Mean ± SEM, ^∗^*p*<0.001, SIH vs. Ctl; *T*-test. **(B,C)** Body weight and food intake evolution. Data are represented as boxplots, Mann–Whitney Rank Sum Test: NS.

Exposure to hypoxia is known to induce physiological adaptations such as an increased erythrocyte production (Michiels, 2004). We therefore evaluated hematocrit at the end of the protocol. We observed an increased hematocrit in mice submitted to SIH compared with control mice (Ctl) (Figure 1A, Mean ± SEM: Ctl : 35, 8 ± 1,4%; SIH: 50, 7 ± 1,8%; *p*<0.001). Body weight and food intake were also measured once a week. No difference between groups was observed in body weight and food intake throughout the experiment (Figure 1B,C).

### Sustained Intermittent Hypoxemia Induced an Ad_mer_ Redistribution While No Modulation in Total Ad_pl_ Level Was Observed

**FIGURE 2:**
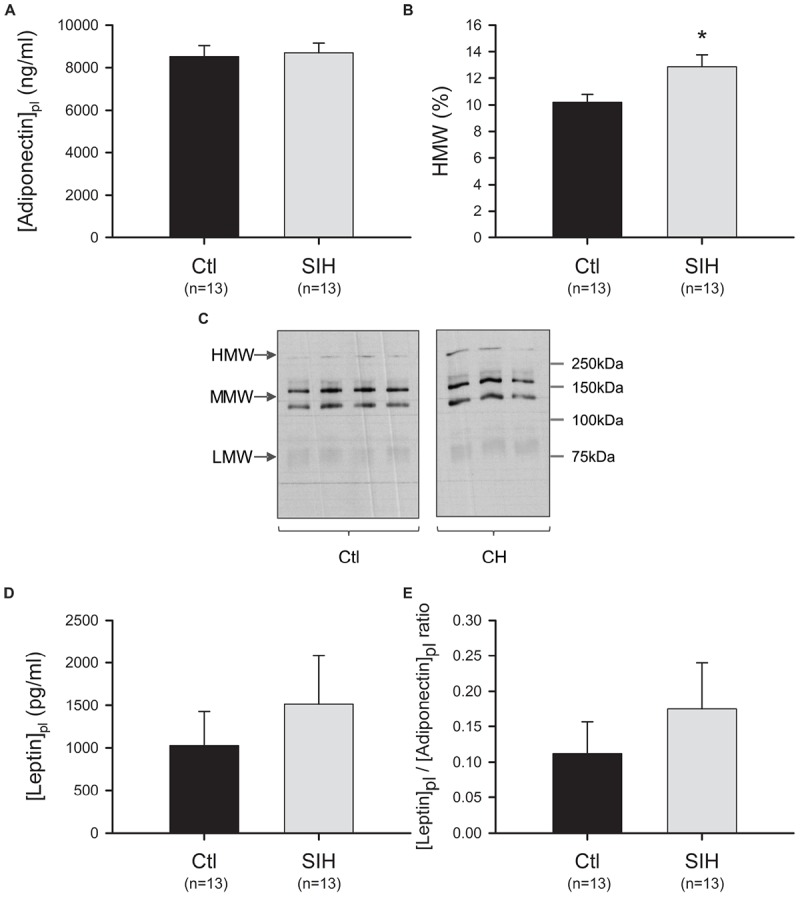
Effect of hypoxemia on Ad and leptin levels **(A)** Ad plasmatic level. Data are represented as mean ± SEM; *T*-test: NS **(B)** Ad_mer_ distribution analysis. The proportion of high (HMW), medium (MMW), and low (LMW) molecular weight Ad forms were determined by using non-denaturant PAGE–SDS followed by Western blot. Ad_mer_/total Ad ratios were obtained after densitometric analysis. Mean ± SEM. ^∗^*p*<0.05, SIH vs. Ctl; *T*-test. **(C)** Ad-mer distribution: representative blots **(D)** Leptin plasmatic level **(E)** Leptin/Ad ratio. Data are represented as mean ± SEM; *T*-test: NS.

As Ad circulates in different multimeric forms, we evaluated whether SIH have an impact on total Ad plasmatic level and on the plasmatic proportion of LMW, MMW, and HMW Ad_mer_. We did not find any modulation of Ad level between groups (Figure 2A). However, an increased proportion of HMW forms was observed in mice exposed to SIH compared with control mice (fold change = 1.26; *p*<0.05) (Figure 2B,C). In parallel, we evaluated leptin plasmatic level and leptin/Ad ratio. No statistical difference was found between groups (Figure 2D,E).

### Sustained Intermittent Hypoxemia Induced a Tissue-Specific Modulation of AdipoR1 and AdipoR2 Protein Abundance Without Change in Their mRNA Expression

**FIGURE 3:**
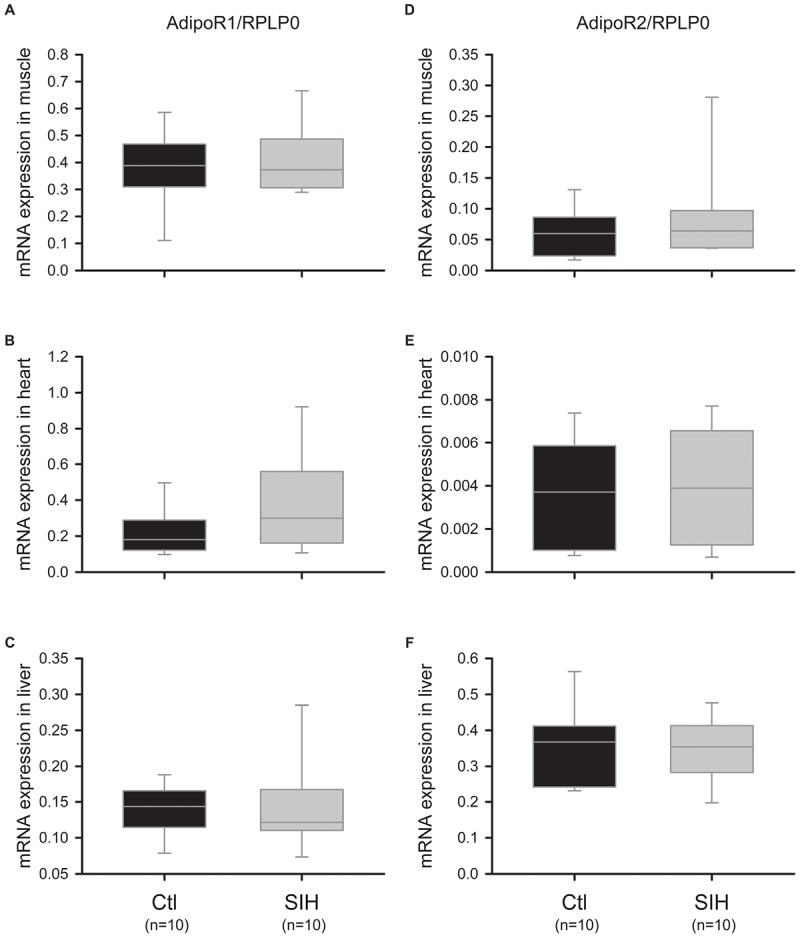
Effect of hypoxemia on AdipoR1/2 expression in peripheral tissues. AdipoR1/2 expression levels were determined by RT-qPCR. **(A–C)** AdipoR1 mRNA expression in muscle **(A)**, heart **(B)**, and liver **(C)**. Data are represented as boxplots (5th and 95th percentiles); Mann–Whitney Rank Sum Test: NS. **(D–F)** AdipoR2 mRNA expression in muscle **(D)**, heart **(E)**, and liver **(F)**. Data are represented as boxplots (5th and 95th percentiles); Mann–Whitney Rank Sum Test: NS.

**FIGURE 4:**
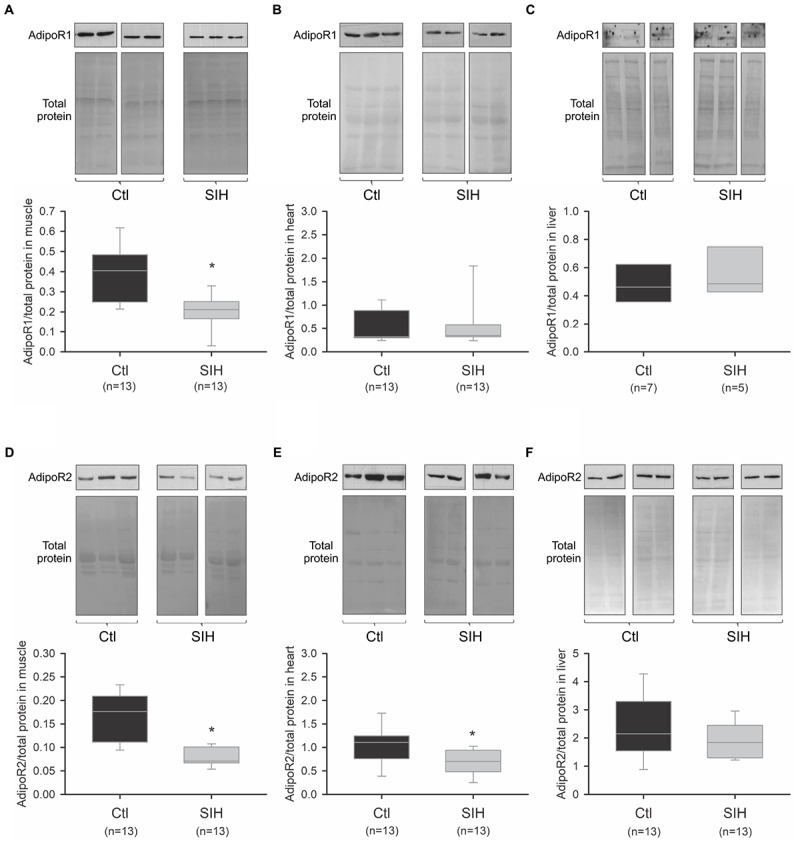
Effect of hypoxemia on AdipoR1 and AdipoR2 protein abundance in peripheral tissues. AdipoR1 and AdipoR2 protein abundance was determined by PAGE–SDS followed by Western blot. The relative quantification was obtained after densitometric analysis. **(A–F)** Representative blots and densitometric analysis of AdipoR1 and AdipoR2 protein abundance in muscle **(A,D)**, heart **(B,E),** and liver **(C,F)**. Data are represented as boxplots (5th and 95th percentiles). ^∗^*p* < 0.05, SIH vs. Ctl, Mann–Whitney Rank Sum Test.

We evaluated whether SIH in mice could modulate AdipoR1 and AdipoR2 mRNA and protein levels in different Ad target tissues (skeletal muscle, heart, and liver). Whereas AdipoR1/2 mRNA expression did not change significantly in skeletal muscle, heart and liver (Figure 3A–F), we observed a tissue-specific modulation of Ad receptors at a protein level (Figure 4). Indeed, a decrease of AdipoR1/2 protein levels was observed in skeletal muscle of SIH mice compared with Ctl mice (AdipoR1: fold change = 0.52; AdipoR2: fold change = 0.40; *p*<0.001). In heart, AdipoR2 protein level was decreased in hypoxemic mice (fold change = 0.63; *p*<0.05) without modification in AdipoR1 abundance. In liver, no modulation of AdipoR1/2 protein levels was observed between groups.

### Mice Submitted to Sustained Intermittent Hypoxemia Exhibited an Improved Glucose Tolerance but Did Not Show Modulation in Their Plasma Lipid Profile

**FIGURE 5:**
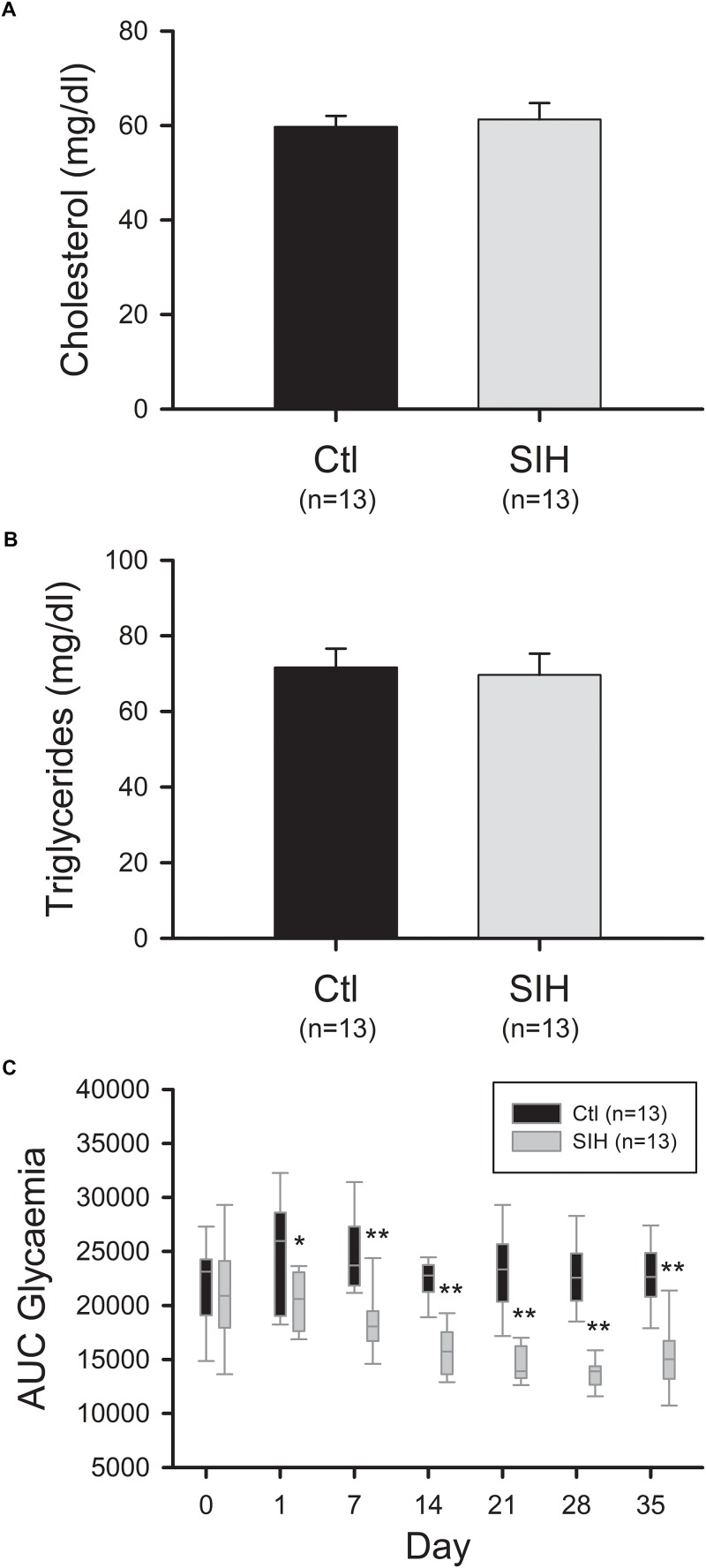
Effect of hypoxemia on blood lipid profile and glucose tolerance. **(A,B)** Cholesterol **(A)** and triglycerides **(B)** plasmatic levels. Plasma lipid concentrations were determined by an enzymatic colorimetric method. Mean ± SEM; *T*-test: NS. **(C)** Modulation of the area under the curve of glycemia over time. Fasted mice were submitted to an intraperitoneal injection of glucose (2 g/kg b.w.). Glycemia was measured before (0) and 30, 60, and 120 min after injection. The area under the curve (AUC) of glycemia was calculated from 0 to 120 min. Data are represented as boxplots (5th and 95th percentiles). ^∗^*p*<0.05, ^∗∗^*p*<0.001, SIH vs. Ctl, Mann–Whitney Rank Sum Test.

As AdipoR protein levels were decreased in hypoxemic mice, we evaluated whether this reduction is associated to a modified lipid profile and glucose tolerance. Circulating triglyceride and cholesterol levels were measured at the end of the protocol and hyperglycemic response once a week. We did not observe any modification of lipid profile between groups (Figure 5A,B). However, a decreased area under the curve (AUC) of glycemia, reflecting an increased glucose tolerance, was observed in SIH mice compared with controls (Figure 5C). This effect was observed from the first day (fold change = 0.79; *p*<0.05) and were maintained until the end of the experiment (fold change = 0.66; *p*<0.001).

## Discussion

Hypoxemia is a critical component of respiratory diseases. Before the development of a persistent hypoxemia, a nocturnal desaturation is observed in COPD patients (Kent et al., 2011; Lewis and O’Halloran, 2016) or in patients with obesity hypoventilation syndrome (Pierce and Brown, 2015). Identifying novel therapeutic targets in these patients appear to be still necessary as they are characterized by multiple troubles leading to an increased cardiovascular risk (Kent et al., 2011). Therefore, studying the modulations of Ad, a cardio-protective hormone, could improve our understanding of the early changes observed in these patients. Consequently, to avoid confounding factors, we evaluated the specific effect of a SIH on Ad_pl_ in a murine model. Ad multimer distribution as well as AdipoR expression were also evaluated to provide a more complete picture of its biological activity.

Regarding the characterization of the murine model, we notice that mice exposed to hypoxemia exhibited an increased hematocrit. This effect is likely mediated by hypoxia-inducible factor 1 (HIF-1), main mediator of cellular adaptation in response to hypoxia (Michiels, 2004; Haase, 2010). Indeed, hypoxia is well known to increase erythrocyte production through an HIF-1-dependant regulation of gene expression such as erythropoietin. As expected, our murine model of hypoxemia is characterized by a secondary erythrocytosis as observed in hypoxemic patients (Kent et al., 2011; El-Korashy et al., 2012).

Ad plasmatic level is not modified in hypoxemic mice in our experimental conditions. However, in previous *in vitro* and *in vivo* studies, Ad expression was reported to be reduced in adipocytes exposed to hypoxia *in vitro* (Hosogai et al., 2007; Ye et al., 2007; Guo et al., 2017; Priyanka et al., 2017) as well as in a rodent model of chronic intermittent hypoxemia (7–21% FiO_2_, 30/30 s, 8 h/day) (Fu et al., 2015; Pan et al., 2015). The difference between these results and our observations could be linked to the used model. In our study, mice were submitted to SIH (10% FiO_2_, 8 h/day during 35 days), a pattern known to induce different pathophysiological adaptations as compared to intermittent hypoxemia (He et al., 2014). Indeed, oxidative stress level and pro-inflammatory cytokine release were higher in rats exposed to IH than in rats submitted to SIH (10% FiO_2_, 8 h/day, 8 weeks) (Zhou et al., 2012; He et al., 2014). As an increased oxidative stress was shown to reduce Ad mRNA level in adipocytes (Furukawa et al., 2004), the absence of Ad level modulation in our model could be partly explained by a distinct level of oxidative stress. In addition to the type of hypoxemia, the duration of exposure is another parameter that could influence Ad circulating levels. Indeed, previous studies did not find any modification of Ad levels in rats submitted to intermittent or continuous hypoxia for 14 days, as well as in mice exposed to hypoxia for 21 days (Chaiban et al., 2008; van den Borst et al., 2013; Briançon-Marjollet et al., 2016). By contrast, after 8 weeks of exposure to continuous hypoxia (FiO_2_: 10%, 24 h/day), Chaiban et al. (2008) observed an increased Ad level in hypoxemic mice.

Whereas no modification in Ad plasmatic levels was found in our hypoxemic mice, we observed an increased proportion of HMW Ad forms. These observations have not been previously investigated in other rodent models of hypoxemia. Although HMW forms were described as the most active forms through its more beneficial activity on insulin sensitivity, inflammation and atherosclerosis (Daniele et al., 2012; Yamauchi and Kadowaki, 2013), no prior study has evaluated the effect of hypoxemia on Ad a multimers. While mechanisms underlying this effect are not known, some hypothesis could be considered. As the different forms of Ad do not interconvert once in the blood stream (Liu and Liu, 2014), the higher proportion of HMW forms in hypoxic condition could be due to an increased synthesis and secretion of these oligomers. Indeed, hypoxia was shown to modulate the expression of the disulfide bond A oxidoreductase-like protein (DsbA-L), a protein involved in Ad multimerization in adipose tissue (Jiang et al., 2013). A modulation of HMW form clearance upon hypoxia could constitute another explanation. Indeed, Ad post-translational modifications such as desialylation were previously found to increase the clearance of this protein (Richards et al., 2010). Therefore, more studies should investigate the effect of hypoxemia on the plasmatic level of Ad multimers. These investigations will provide us a better understanding of the effect of an increased HMW forms on several processes involved in the progression of hypoxemia-associated diseases.

The redistribution of Ad multimers observed in our study was associated to a tissue-specific modulation of AdipoR abundance. Indeed, AdipoR1 and AdipoR2 protein abundance were reduced in sketelal muscle of hypoxemic mice. In the heart, AdipoR2 abundance was also reduced in hypoxic conditions while AdipoR1 protein level did not change. No modulation of AdipoR abundance was observed in liver between groups. Previous studies have also found a regulation of AdipoR expression depending on the target tissue (Huang et al., 2006; Pierard et al., 2016). However, to our knowledge, there is no study concerning the effect of hypoxemia on AdipoR abundance *in vivo*. As it is well known that distinct cells exhibit different sensitivity to hypoxia and different physiological adaptations *in vitro* (Michiels, 2004; McNamee et al., 2013), a tissue-specific regulation of AdipoR protein levels was not surprising. An hypothesis is that AdipoR protein variations could be influenced by HIF-1α basal level and regulation, which vary among tissues (Stroka et al., 2001; Michiels, 2004). Moreover, Stroka et al. (2001) found that nuclear HIF-1α protein level was detected in liver of mice only when they were exposed to a severe hypoxemia (6% FiO_2_). The absence of modulation of AdipoR abundance in liver in our murine model of hypoxemia (10% FiO_2_) may therefore be related to a less severe sensitivity. Moreover, as we did not detect any modification of AdipoR expression at the mRNA level, our results suggest that hypoxemia could modulate AdipoR protein abundance through post-transcriptional or post-traductional modifications. Indeed, the microRNA 218 was previously found to decrease AdipoR2 protein level (Du et al., 2015) and reported to be induced in hypoxic condition (Liu et al., 2017). Another mechanism could be an increased lysosomal degradation of AdipoR. Such regulation of AdipoR protein level was reported in previous studies (Ding et al., 2009; Almabouada et al., 2013), but its occurence upon hypoxemia remains to be elucidated.

In our study, we also observed that hypoxemia exerts different effects on AdipoR1 and AdipoR2 in the same tissue. In muscle, the abundance of both AdipoR was decreased in hypoxic conditions, while only a reduced AdipoR2 protein level was observed in the heart. The differential regulation of AdipoR1 and AdipoR2 was previously shown in other studies and could have different consequences as those adiporeceptors exhibit divergent functions.

Because of the know role of AdipoR-dependant pathways on lipid metabolism, we evaluated plasma lipid profile (triglycerides and cholesterol levels) in hypoxemic mice and we did not observe any modification as compared with controls. No other study has been conducted to evaluate lipid profile upon long-term SIH but an increased triglyceride and cholesterol levels was reported in mice exposed to 4 or 12 weeks of intermittent hypoxia (Savransky et al., 2007; Drager et al., 2013; Yao et al., 2013). Interestingly, Holland et al. (2017) found recently that transgenic mice overexpressing AdipoR in the liver exhibited lowered basal serum triglycerides than wild-type mice, suggesting an impact of hepatic AdipoR expression on this parameter. In our experimental conditions, hepatic abundance of AdipoR did not differ between groups.

Regarding the hyperglycemic response, hypoxemic mice exhibited an increased glucose tolerance compared with control mice. This effect was observed from day 1 and was maintained until the end of the protocol. It is well established that hypoadiponectinemia is associated to insulin resistance in humans (Addy et al., 2003). Indeed, Ad improves insulin sensitivity by an increased muscle glucose uptake, a decreased hepatic gluconeogenesis as well as by an increased fatty acid oxidation in liver and skeletal muscle (Kadowaki et al., 2006; Lustig et al., 2012). The increased glucose tolerance observed in our study could therefore be induced by the increased plasmatic levels of HMW forms, which exhibited a more important action on insulin sensitivity (Wang et al., 2008; Yamauchi and Kadowaki, 2013). However, as observed in our study, the increased HMW forms proportion was reported to be also associated to a decreased abundance of AdipoR1 and AdipoR2 in skeletal muscle, therefore inducing a phenomenon called “Ad resistance.” Previous studies found that the stimulatory effect of Ad on muscle fatty acid oxidation and insulin-stimulated glucose transport could be impaired in skeletal muscle, at least partly due to a downregulation of AdipoR expression (Mullen et al., 2007, 2009; Sente et al., 2016). Moreover, Yamauchi et al. found that the beneficial effects of Ad on glucose levels was abolished in AdipoR1 and AdipoR2 double-knockout mice. In addition, an improvement of glucose tolerance in hypoxemic mice was previously described in other studies (Gamboa et al., 2011; Thomas et al., 2017). While the exact mechanism remains controversial, various mechanisms were suggested such as an increased AMPK activity, GLUT4 translocation (Thomas et al., 2017), or Akt activation which leads to an increased glycogen synthesis (Gamboa et al., 2011). As the anti-diabetic effects of Ad include the contribution of all of these components, we could hypothesize that, although the reduced AdipoR abundance could interfere with the beneficial effects of Ad, the improved glucose tolerance may be partly attributed to an increased levels of HMW forms. This hypothesis is in accordance with the absence of modulation of AdipoR protein levels in liver, suggesting that HMW forms could improve glucose tolerance through its beneficial effects on this tissue. However, as HIF-1 is well known to increase enzyme expression of the glycolytic pathway (Goda and Kanai, 2012) as well as glucose uptake in muscle by increasing GLUT4 translocation (Sakagami et al., 2014), we are not able to exclude that other mechanisms could contribute to the improved glucose tolerance observed in our model. Indeed, Serebrovska et al. (2017) observed an increased expression of HIF-1 target gene and an improved glucose tolerance in subjects with prediabetes (*n* = 11) exposed to intermittent hypoxia (12/21% FiO_2_, four cycles of 5/5 min during 1 month). They suggested a beneficial effect of intermittent hypoxia on glucose tolerance, but the strict lifestyle environment they have applied could have interfered with this relation. Further studies are needed to better understand the impact of SIH on glucose homeostasis.

## Conclusion

In conclusion, SIH in mice modulated Ad_mer_ distribution with an increased proportion of Ad HMW forms and also induced a tissue-specific modulation of AdipoR protein abundance. Indeed, whereas hypoxemia, *per se*, did not modify AdipoR expression in peripheral tissues, it causes a decreased AdipoR1/2 protein abundance in skeletal muscle and a reduced AdipoR2 protein level in heart. Although molecular mechanisms underlying hypoxemia-mediated effects have to be further investigated, modulations of Ad forms and receptors suggested modifications of Ad-dependant pathways in pathological conditions associated to hypoxemia. Investigation of those parameters could constitute useful complementary criteria for risk stratification and to identify novel therapeutic targets to counteract the development of metabolic and cardiovascular co-morbidities in respiratory diseases.

## Author Contributions

MP carried out the animal experimentation and molecular studies, participated in the design of the study, drafted the manuscript, and performed the statistical analysis. SC participated in the animal experimentation as well as the design and coordination of the study. KZB participated in the conception of the study and in its design and coordination. AL and AT conceived the study, and participated in its design and coordination, helped to draft the manuscript, and to perform the statistical analysis. All authors read and approved the final manuscript.

## Conflict of Interest Statement

The authors declare that the research was conducted in the absence of any commercial or financial relationships that could be construed as a potential conflict of interest.
