# Supplementary material for: Sustained Intermittent Hypoxemia Induces Adiponectin Oligomers Redistribution and a Tissue-Specific Modulation of Adiponectin Receptor in Mice
Source: Front Physiol. 2019 Feb 8;10:68. doi: 10.3389/fphys.2019.00068 (PMC6376175; doi:10.3389/fphys.2019.00068)
Supplement: Supplementary file 1 [file Table_1.DOCX]

**Table S1. Primer sequences used in RTqPCR analysis**

| Target genes | Primer sequences |
| --- | --- |
| AdipoR1 | Forward: 5’ –TCTTCGGGATGTTCTTCCTGG– 3’  Reverse: 5’ –TTTGGAAAAAGTCCGAGAGACC– 3’ |
| AdipoR2 | Forward: 5’ –CCTTTCGGGCCTGTTTTAAGA– 3’  Reverse: 5’ –GAGTGGCAGTACACCGTGTG– 3’ |
| RPLP0 | Forward: 5’ –GGGCATCACCACGAAAATCTC– 3’  Reverse: 5’ –CTGCCGTTGTCAAACACCT– 3’ |
